# Regulation of emergency granulopoiesis during infection

**DOI:** 10.3389/fimmu.2022.961601

**Published:** 2022-09-05

**Authors:** Sagar Paudel, Laxman Ghimire, Liliang Jin, Duane Jeansonne, Samithamby Jeyaseelan

**Affiliations:** ^1^ Center for Lung Biology and Disease, Louisiana State University (LSU) School of Veterinary Medicine, Baton Rouge, LA, United States; ^2^ Department of Pathobiological Sciences, Louisiana State University (LSU) School of Veterinary Medicine, Baton Rouge, LA, United States; ^3^ Section of Pulmonary and Critical Care, Department of Medicine, LSU Health Sciences Center, New Orleans, LA, United States

**Keywords:** granulopoiesis, infection, transcription factors, cytokines, chemokines

## Abstract

During acute infectious and inflammatory conditions, a large number of neutrophils are in high demand as they are consumed in peripheral organs. The hematopoietic system rapidly responds to the demand by turning from steady state to emergency granulopoiesis to expedite neutrophil generation in the bone marrow (BM). How the hematopoietic system integrates pathogenic and inflammatory stress signals into the molecular cues of emergency granulopoiesis has been the subject of investigations. Recent studies in the field have highlighted emerging concepts, including the direct sensing of pathogens by BM resident or sentinel hematopoietic stem and progenitor cells (HSPCs), the crosstalk of HSPCs, endothelial cells, and stromal cells to convert signals to granulopoiesis, and the identification of novel inflammatory molecules, such as C/EBP-β, ROS, IL-27, IFN-γ, CXCL1 with direct effects on HSPCs. In this review, we will provide a detailed account of emerging concepts while reassessing well-established cellular and molecular players of emergency granulopoiesis. While providing our views on the discrepant results and theories, we will postulate an updated model of granulopoiesis in the context of health and disease.

## Introduction

Ever since Metchnikoff first observed phagocytosis of bacteria in white blood cells in 1882 (1), we have been fascinated by neutrophils as they have taken the spotlight of the innate immune system. Neutrophils play a central role in host defense against bacterial, viral, and fungal infections. Patients with neutropenia and clinical disorders (severe congenital neutropenia, leukocyte adhesive deficiency, and chronic granulomatous diseases) suffer from severe infections, which highlights the important role of neutrophils in host immunity. On the flip side, uncontrolled neutrophil extravasation may lead to excessive collateral inflammatory damage found in conditions such as acute respiratory distress syndrome.

Neutrophils are short-lived and therefore the human body generates about 0.5-1 x 10^11^ neutrophils per day in the bone marrow (BM) to maintain the circulating pools during steady-state conditions (2). Neutrophils develop from the dormant but self-renewing hematopoietic stem cells (HSCs), which reside in a specialized BM niche and represent the topmost cells in the hierarchy of the hematopoietic system (3, 4). Long-term HSCs first differentiate to short-term HSCs (ST-HSCs), which then give rise to multi-potent but non-renewing differentiated cells, multi-potent progenitors (MPPs). Further downstream, MPPs can differentiate into oligopotent common myeloid progenitors (CMPs) and common lymphoid progenitors (CLPs). Then, CMPs advance as either granulocyte monocyte progenitors (GMPs) giving rise to neutrophils and macrophages or megakaryocyte/erythrocyte progenitor (MEP) giving rise to platelets and erythrocytes. HSC, its niche and lineage specifications in health and disease has been the recent subject of excellent reviews published elsewhere (5–7). A comprehensive review on neutrophil ontogeny and biology with specific emphasis on the differences between neonatal and adult neutrophil populations has been recently offered by Lawrence et al. (8).

**Figure 1:**
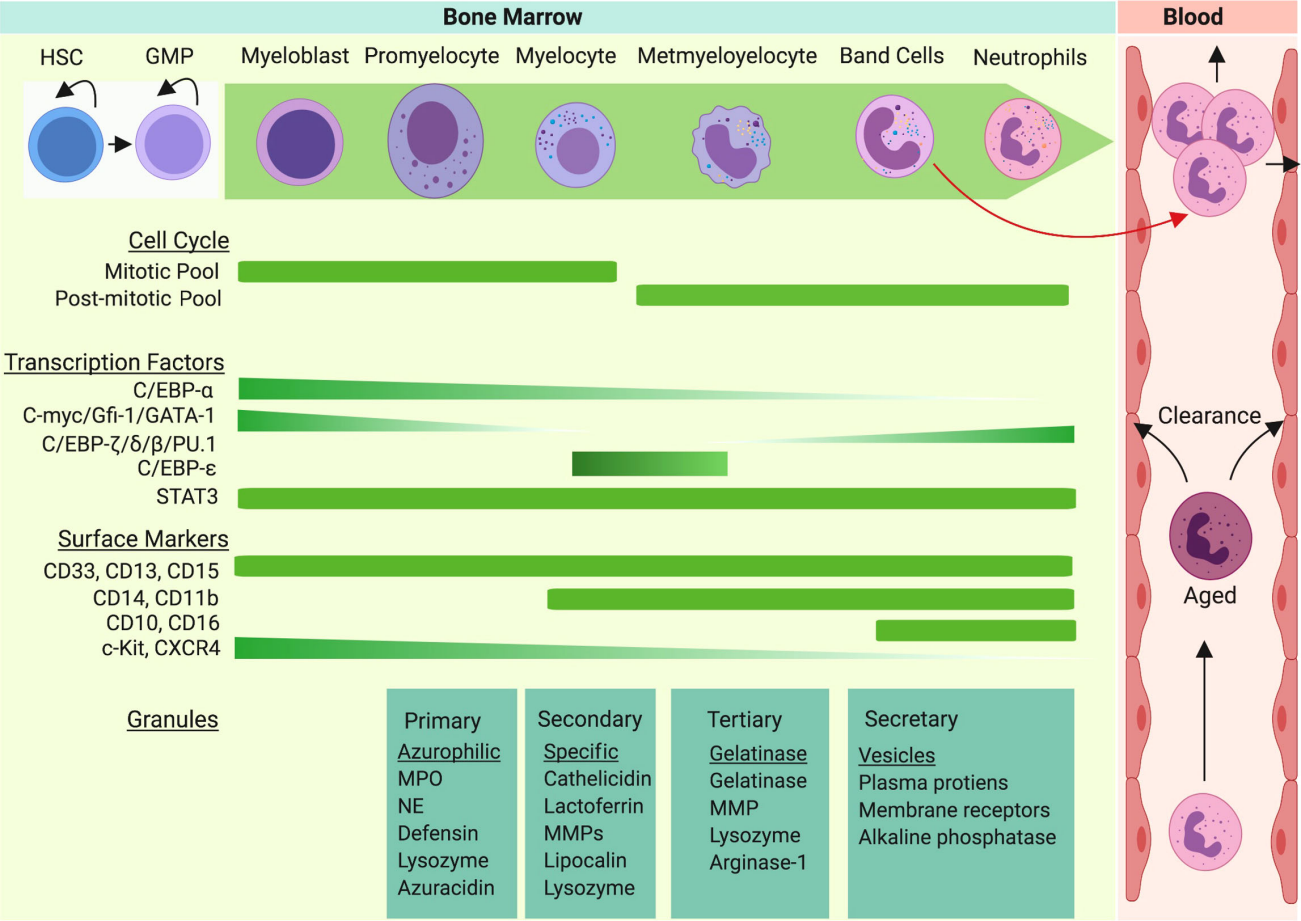
Neutrophil granulopoiesis is a hierarchical program of the hematopoietic system. Granulopoiesis, the process of neutrophil generation and maturation, is characterized by sequential development of distinct granules, surface proteins, and nuclear segmentation. Homeostatic granulopoiesis starts with generation of myeloblasts and promyelocytes (rich in primary azurophilic granules) which depends on expression of GATA-1, C/EBP-α, Gfi-1, and c-Myc genes. The development of secondary specific granules appears in myelocytes (last proliferative stage). C-Myc and C/EBP-ϵ contribute to formation of secondary granules. The development of metamyelocytes with gelatinase granules marks terminal differentiation of neutrophil granulopoiesis. As expression of C/EBP-β, C/EBP-δ, and C/EBP-ζ are upregulated in terminal neutrophil stages, gelatinase granules and secretary vesicles appear and nuclear segmentation intensifies. During emergency granulopoiesis, C/EBP-β takes over the process and an excess amount of neutrophils are generated and released to the bloodstream along with immature neutrophils (band cells).

During hematopoietic stress such as systemic infections or physical insults, neutrophils are used up in larger quantities and the demand for neutrophils in the peripheral blood increases by several folds higher than steady state. The hematopoietic system rapidly senses this neutrophil demand and integrates these pathogen signals to activate dormant HSCs to proliferate and differentiate into neutrophil progenitors (**Figure 1**). This distinct program of accelerated *de novo* production of neutrophils from amplification of neutrophil progenitors is known as ‘*emergency granulopoiesis*’, which is usually a protective hematopoietic immune response to fatal infection. This process of differentiation and then amplification of multipotent HSCs into mature, segmented neutrophils is highly sophisticated and governed by a complex network of molecular and cellular players (**Figure 1**). Clinical manifestations of patients with heightened emergency granulopoiesis are elevated levels of acute phase proteins, leukocytosis, neutrophilia, and appearance of immature myeloid precursor cells (left shift).

In this review, we provide a detailed examination of the cellular and molecular players involved in activation of emergency granulopoiesis, the molecular messengers that integrate pathogen signals into HSC activation, proliferation and differentiation into neutrophils, and regulatory molecular and environmental mechanisms that control emergency granulopoiesis in the context of infectious and inflammatory conditions.

## Granulopoiesis: Steady state vs emergency

As neutrophils are mitotically inactive and relatively short lived, their counts in circulation during physiological conditions are constantly replenished through a process known as ‘*steady-state granulopoiesis*’. Localized mild-grade bacterial infections do not invoke strong hematopoietic pressure, as the host rapidly resolves infections, limiting their systemic dissemination. Consequently, HSCs do not sense the demand and BM granulopoiesis remains unaltered from the steady state. However, overwhelming systemic infections put much stronger stress to hematopoietic systems, as large quantities of neutrophils are lost in an attempt to limit dissemination. To meet urgent needs of depleting neutrophils, the BM hematopoietic system rapidly switches steady-state granulopoiesis to a well-coordinated process of neutrophil generation in a large scale that involves activation and proliferation of myeloid progenitors known as ‘*emergency granulopoiesis*’. Studies suggest BM niche adjust to allow space for the heightened kinetics and magnitude of emergency granulopoiesis at the expense of erythropoiesis and lymphopoiesis, as inflammatory signals are shown to reduce the expression of lymphoid expansion and retention signals in BM (9–11). Besides systemic infections, iatrogenic conditions such as the irradiation or chemotherapy-induced myeloablation results in profound neutropenic conditions (12, 13) and can trigger emergency granulopoiesis to replenish neutrophil homeostasis. Despite their differences in stimuli, many cellular and molecular events of granulopoiesis are shared. However, accumulating evidence shows that infection-induced emergency granulopoiesis may have a major evolutionary impact on the response of the hematopoietic system to stress. This notion of evolution of the hematopoietic system in response to stress became clearer as HSCs were shown to circulate, sense, and respond to pathogen recognition (14–16). With the discovery of CCAAT/enhancer-binding protein (C/EBP) in the regulation of granulopoiesis (9, 17, 18), we now know that emergency granulopoiesis is differentially regulated at the transcriptional level from steady state.

## Pathogen sensing system in emergency granulopoiesis

**Figure 2:**
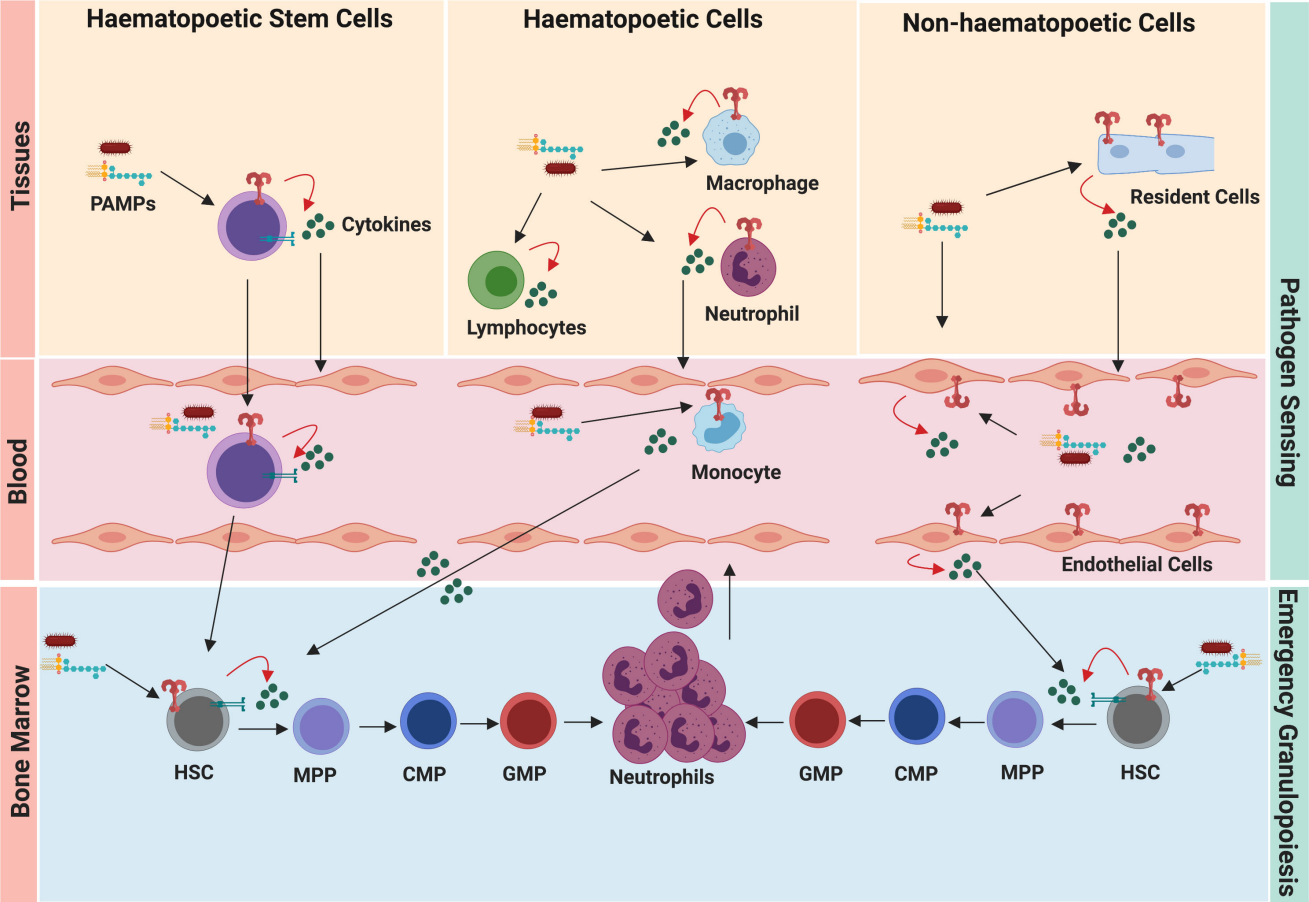
Pathogen sensing system of emergency granulopoiesis. During infection and inflammation, the hematopoietic cells, resident cells, and circulating HSCs sense disseminating pathogens and danger signals. Pathogen-induced granulopoiesis heavily depends upon TLR-signaling, which translates pathogen signals into molecular cues of granulopoiesis. TLR engagement on these cells results in the secretion of several granulopoietic growth factors (such as G-CSF, IL-6, CXCL1), which initiate signaling cascades in HSCs and neutrophil progenitors, thereby activating emergency granulopoiesis. Expression of TLRs by HSCs and endothelial cells facilitates more direct ways of initiating granulopoiesis compared to indirect activation of immune cells at the site of infection/inflammation.

The hematopoietic system usually responds to infection by enhancing cellular output, but how local/systemic infections are sensed and subsequently messaged to the BM is not very clear. Given that the granulopoietic BM niche is spatially disconnected from the site of pathogen entry, it was long believed that activation of emergency granulopoiesis is an indirect process and relies on sensing of pathogen by germ-line encoded pathogen recognition receptors (PRRs) by immune and resident cells. Toll-like receptors (TLRs)-expressing cells are first to detect pathogens or their products (**Figure 2**). These cells do not generate neutrophils by themselves but secrete hematopoietic cytokines (such as G-CSF, IL-6) which indirectly influence differentiation and proliferation of neutrophil precursors (19). However, recent reports show that HSPCs can express pattern recognition receptors (11, 20, 21) as well as multiple traffic molecules, and egress and re-enter the BM niche (14, 15), thereby suggesting immediate and direct activation of emergency granulopoiesis by these precursor cells (**Figure 2**). In the following section, we will dive deeper into understanding how the hematopoietic system has evolved to sense pathogens and to rapidly message the bone marrow niche.

## HSPCs

Immature HSCs and early progenitors express PRRs and initiate granulopoiesis directly as they can actively participate in recognition of pathogen-derived products (20–22). Nagai et al. were first to demonstrate that HSCs (Lin^-^c-Kit^+^ subset) express TLR2, TLR4 and their co-receptors (MD-2, CD14) and GMPs (Lin^-^c-Kit^+^Sca-1^-^CD34^+^FcγR^+^subset) express TLR2 (20). Furthermore, *ex vivo* stimulation of HSCs with TLR2 agonist (Pam_3_CSK_4_) or with TLR4 agonist (LPS) drove MyD88-dependent but cytokine-independent differentiation to produce myeloid lineage cells (20). Immediately, Massberg et al. revealed that lymph borne HSPCs originate in the BM and possess extensive multi-lineage potential even at peripheral organs when encountering pathogens (15). Specifically, these HSPCs are shown to proliferate to myeloid cells within the kidney when implanted under the kidney capsule in response to TLR agonist (15). Human CD34^+^ HSPCs also express functional TLRs and whose ligation with agonists induces the differentiation into myeloid cells without addition of cytokine, in some cases, at the expense of lymphopoiesis (23–25). Since then, numerous studies have shown TLR ligation as an important step in HSC differentiation to myeloid cells (11, 26–28). The functional consequence of TLR engagement was further elucidated when an elegant study demonstrated that LPS-stimulated HSPCs secrete IL-6 and that HSPCs derived from IL-6^-/-^ mice failed to differentiate to myeloid cells under neutropenic conditions achieved with the treatment of either chemotherapy (5-fluorouracil) or irradiation (29). Besides TLR2 and TLR4, a recent study demonstrated that TLR7 signaling drove type I IFN-dependent myeloid differentiation of CMP *in vitro* and peripheral expansion of monocytes *in vivo* (30). A recent study highlighted that the *in vivo* response of HSC to pathogens is much more complex and redundant, as MyD88/Triff^-/-^ mice or C3H/HeJ (loss of function mutation for TLR4) mice had normal expansion of HSPCs during *S. aureus* infection (31). Collectively, these studies clearly demonstrate that the hematopoietic system has evolved to detect and respond to pathogens directly through TLRs and these encounters are all geared to HSC differentiation, amplification of progenitors, and generation of progeny cells at the BM niche or at the site of infection in some cases.

## Hematopoietic and non-hematopoietic cells

A long prevailing hypothesis is that emergency granulopoiesis is indirectly activated by hematopoietic and resident cells *via* inflammatory mediators. Tissue resident macrophages and circulating monocytes are equipped with PRRs, sense pathogens at the site of entry, and release massive amounts of granulopoietic cytokines (such as G-CSF, GM-CSF, IL-6) during local or systemic infection. With the discovery of IL-17A-producing innate lymphoid cells and regulatory properties of neutrophils (32), we speculate that monocytes and macrophages are not the sole players. Although there is lack of careful *in vivo* experimentation proving direct roles of these cells in emergency granulopoiesis, the abundance of data suggests their role is largely mediated by cytokine and growth factors which can activate HSC differentiation, regulate cell survival, and generate lineage committed myeloid cells. Experiments with antibody-mediated depletion have broadened our knowledge of these cells but careful interpretation of data should be made, as the observation could be a positive feedback granulopoiesis. For instance, anti-Ly6G depleted neutropenic mice or LysM^Cre/wt^ Mcl-1^f/f^ mice (lacking mature neutrophils) during steady state elicited elevated levels of G-CSF resulting in enhanced HPSC proliferation and GMP differentiation (33). Soon after, Bugl et al. (34) not only confirmed the enhanced G-CSF and HSC expansion in neutropenic mice but also showed G-CSF-mediated feedback granulopoiesis was dependent on BM neutrophil mass. In contrast to popular belief, using clodronate liposome or reciprocal TLR4^-/-^ chimera, Boettcher et al. were first to reveal that selective TLR4 expression within the hematopoietic compartment failed to induce emergency granulopoiesis and neutrophilia during systemic LPS injection (35). TLR4 signaling in non-hematopoietic but not in the hematopoietic compartment was an absolute requirement for LPS-induced, G-CSF-mediated granulopoiesis (35). In another elegant work, Boettcher et al., using endothelial cell (EC) specific MyD88^-/-^ mice in bone marrow chimeras, demonstrated that EC-intrinsic MyD88 signaling dominantly contributed to G-CSF production and induced GMP expansion and neutrophil generation after *in vivo* LPS or *Escherichia coli* challenge (36). As the highest G-CSF expression was noted in BM EC after LPS challenge, it is convincing that vasculature lining ECs could be an important layer of local signal to the BM niche to initiate granulopoiesis.

## NOD-like receptors

Until recently, the role of intracellular NLRs in hematopoiesis was elusive. Burberry et al. showed that NOD1 and NOD2 cooperate with TLR4 receptors to lower CXCL12 in BM and induce G-CSF, thereby mobilizing HSC to give rise to neutrophils and monocytes during *E. coli* infection (37). Furthermore, NLRP3 inflammasome regulates HSC mobilization to peripheral blood through IL-1β and IL-18 production following administration of G-CSF and AMD3100 (38). However, genetic ablation of caspase-1/11 in neonatal mice resulted in the enhanced emergency myelopoiesis possibly through expansion of BM and splenic HSC and higher G-CSF/M-CSF levels, implicating an NLR/caspase/IL-1β axis in granulopoiesis (39). Mice deficient in NLRC4 and NLRP6 have enhanced neutrophil recruitment and elevated levels of hematopoietic cytokines during pulmonary and systemic infection (40–42), but whether these mice also present granulopoietic defects in BM was not explored. More directed studies are needed to identify if these intracellular sensors are actually a class of negative regulator of TLR-mediated granulopoiesis.

## Molecular translation of pathogen signals into granulopoiesis

**Figure 3:**
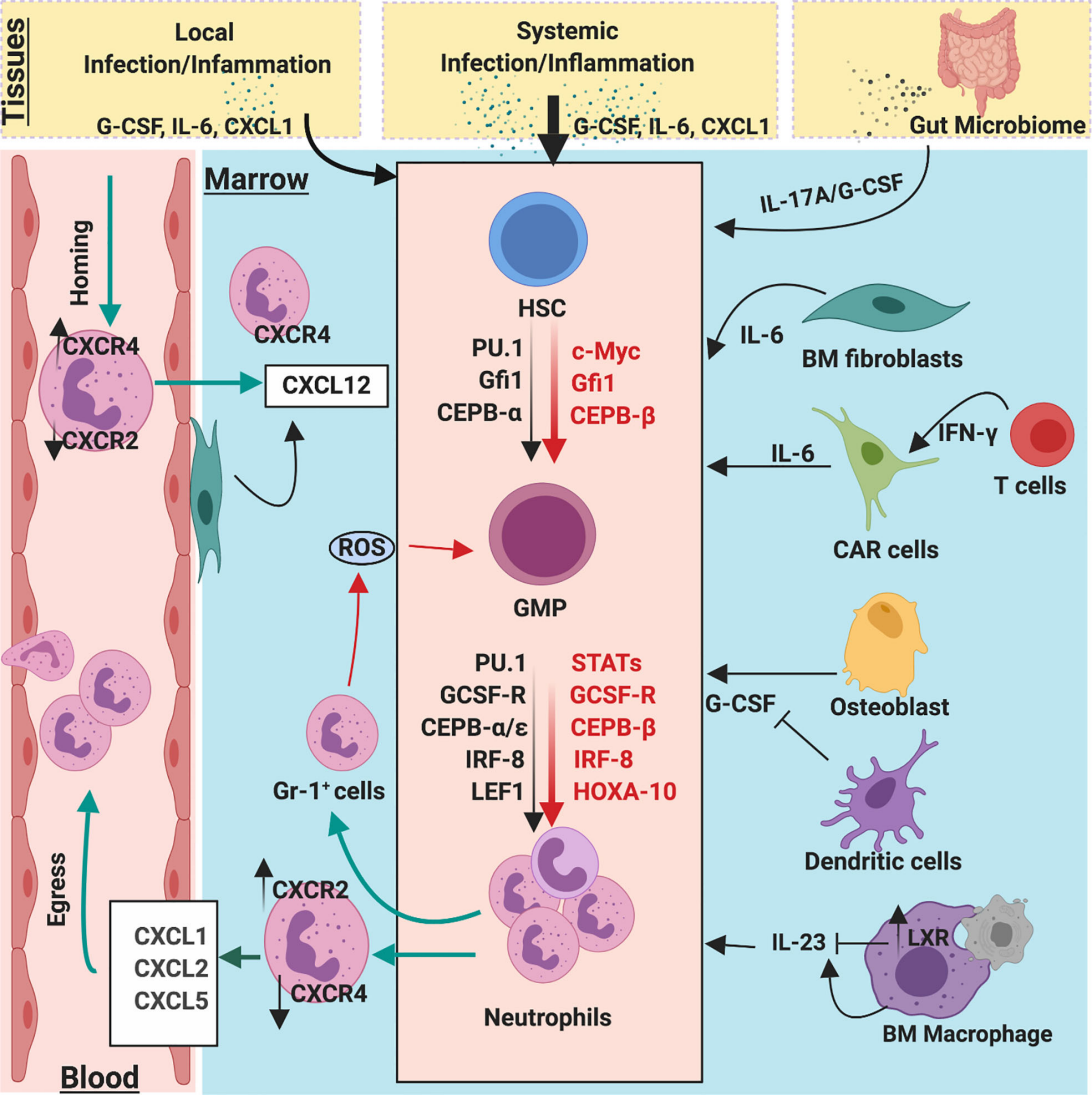
Molecular and cellular regulators of emergency and homeostatic granulopoiesis. Granulopoiesis is a tightly regulated process by several molecular and cellular players. During the steady state, C/EBPα is a primary transcription factor that regulates proliferation of neutrophil progenitors by limiting the expression of cell cycle gene such as c-Myc, CDK2, and CDK4. However, during emergency granulopoiesis, C/EBPβ directly replaces C/EBPα at the *MYC* promoter and expedites c-Myc expression to enhance the generation of neutrophils. Robust C/EBPβ activity relies on strong activation of JAK/STAT pathways by G-CSF, which is massively released during infection/inflammation. During steady state, clearance of apoptotic neutrophils by macrophages activates LXR expression, which downregulates IL-23 secretion, thereby limiting the activity of G-CSF/IL-17A. Bone marrow stromal fibroblast and CXCL12 abundance reticular (CAR) cells also secrete IL-6 which regulate granulopoiesis. Additionally, ROS release from BM GR-1^+^ cells also regulate proliferation and differentiation of GMPs into neutrophil lineages. Gut microbiome provides PAMPs to activate and regulate steady-state and emergency granulopoiesis through the IL-23/G-CSF/IL-17A axis.

The hematopoietic system must translate pathogen sensing into the process of emergency granulopoiesis (**Figure 3**). Although little emerging evidence suggests HSPCs can respond to direct pathogen sensing, a vast number of studies unequivocally demonstrate that the coordinated action of myeloid growth factors, cytokines, chemokines, and transcription factors mediate emergency granulopoiesis. Recent evidence reveals these mediators can instruct lineage choice for uncommitted HSPCs rather than just enhancing proliferation and survival of the precursor. In this section, we dissect how these myeloid growth factors mediate HSPC differentiation, lineage choices, proliferation, and survival of myeloid progenitors during emergency granulopoiesis.

## Colony stimulating factors

Myeloid growth factors such as G-CSF (and its receptor G-CSFR), GM-CSF, M-CSF are the best-studied colony stimulating factors in context of granulopoiesis. Hematopoietic cells, including HSPCs, are known to express receptors for these cytokines (11). *In vivo* daily administration of G-CSF or GM-CSF with stem cell factor resulted into neutrophilia and a synergistic GMP expansion in bone marrow (43). GM-CSF stimulates the expansion of myeloid lineage cells both *in vivo* and *in vitro* (44, 45). Daily *in vivo* administration of M-CSF expanded the number of blood monocytes and resident macrophages in spleen and liver (46). Mice deficient in G-CSF (*Gcsf^-/^* ^-^) and G-CSFR (*Gcsfr^-/-^*) displayed a diminished level of peripheral neutrophils and a corresponding decrease in neutrophil progenitors in the BM niche during steady state (47, 48). Exogenous administration of G-CSF into uninfected *Gcsf^-/-^* mice augmented the level of circulating neutrophils (47). However, the role of GM-CSF in baseline appeared redundant as mice deficient in GM-CSF (*Gmcsf^-/-^*) showed no major defect in steady-state granulopoiesis and maintained normal pools of myeloid cells and their precursors in blood, marrow, and spleen (49). However, the usage of these mice in infection-induced granulopoiesis has yielded some discrepant results. Earlier reports demonstrated that both *Gcsf^-/-^ and Gmcsf^-/-^* mice had impaired emergency granulopoiesis and host defense against *Listeria monocytogenes* infection (47, 50). In contrast, emergency granulopoiesis in *Gcsf^-/-^* mice were not dampened but indistinguishable from control mice when challenged with *Candida albicans* (51). These *Gcsf^-/-^* mice have a strikingly high level of IL-6 but mice deficient in both G-CSF and IL-6 also developed neutrophilia and BM expansion of GMPs (51). Furthermore, mice deficient in GM-CSF (*Gmcsf^-/-^*) have reduced circulating pools of hematopoietic cells but surprisingly displayed subtle changes in BM precursors during *Mycobacterium avium* infection (52). Additionally, mice deficient in all three colony stimulating factors (G-CSF, GM-CSF, M-CSF) have reduced circulating myeloid cells during the resting state, but they were surprisingly able to mount emergency granulopoiesis during thioglycolate-induced peritonitis (53). Accumulating evidence shows intriguing functional consequences of these cytokines on cellular targets. Mechanistically, G-CSF acts as a proliferative signal for HSCs but impairs renewal ability, which is dependent on TLR signaling and host microbiome (54). Additionally, G-CSF disrupts the HSC niche resulting in their mobilization (55), suggesting a G-CSF-induced defect in repopulation could be in fact increased HSCs mobilization from the BM niche. Using long-term bio-imaging observations at the single-cell level, Rieger et al. (56) demonstrated that bipotent GMPs responded with generation of exclusively mature neutrophilic granulocytic or monocytic cells in response to G-CSF and GM-CSF. More insights on the instructive action of cytokines came into light as Mossadegh-Keller et al. (57) utilized time-lapse imaging and single cell gene expression analysis to reveal that M-CSF directly stimulates HSCs to activate transcription factor PU.1, which promotes differentiation into the myeloid cell lineage. Additionally, Jack et al. (58) have demonstrated that M-CSF targets ERK to elevate c-Fos and phospho-C/EBPalpha whereas G-CSF influences SHP2 activity in marrow progenitors to impact the lineage specification. Epstein-Barr virus infected humanized NOG mice demonstrate increased granulopoiesis along with elevated levels of GM-CSF (59). Finally, Bacillus Calmette–Guérin (BCG) vaccination-induced rapid protection from neonatal sepsis is mediated by G-CSF-induced emergency granulopoiesis (60).

## CXC and CC-chemokines

Although chemokines and their receptors have long been described on their ability to recruit leukocytes, recent reports reveal that chemokine systems are increasing involved in the differentiation, proliferation, and mobilization of hematopoietic progenitors (61, 62). Copiously produced by the various cells of BM niche (63), CXCL12 (or SDF1) and its receptor CXCR4 axis is a representative homeostatic chemokine system primarily regulating HSC retention (64). Mice deficient in CXCL12 or CXCR4 displayed a virtual absence of myelopoiesis in BM, reduced number of myeloid cells in fetal liver, and impaired B-lymphopoiesis (65, 66). Recently, CXCL12 has been shown to regulate mitochondrial respiration in early HSCs, which are critical for maintaining their undifferentiated state (67). CCL3, a CC-chemokine that has long been implicated for hematologic malignancies (68), has been shown to enhance myelopoietic activity for mature progenitors or suppress myelopoietic activity for more immature progenitors (69). Using CCR1-deficient mice, CCL3 signaling through CCR1 receptor was shown to play a dominant role for the proliferation but not suppression of lineage-committed myeloid progenitors (70). In contrast, mice lacking CCL3 (*Ccl3^-/-^*) have profound increases in HSPCs and immature myeloid progenitors but reduced numbers of committed myeloid progenitors without altering BM microenvironmental populations (71). In contract to CXCL12/CXCR4 and CCL3/CCR1, CCR2 and its ligands (CCL2/12/13) have long been known to have myelosuppressive effects (72). Using RFP-CCR2 reporter mice, the profound expression of CCR2 was observed in neutrophils (73). Additionally, mice lacking CCR2 displayed increased sequestration of all subsets of myeloid cells in BM and their corresponding reduced numbers in blood and spleen (73). Recently, another CC-chemokine, CCL5/RANTES, has been demonstrated to influence the BM niche cell subtypes and lineage skewing phenotype (74). Indeed, mice lacking CCL5 (*Ccl5^-/-^*) displayed reduced levels of myeloid-biased HSCs and myeloid progenitors but concomitant increases in lymphoid-biased HSCs and T cells progeny (74). However, the retroviral expression of CCL5 in BM progenitors or a brief *ex vivo* stimulation of HSCs with CCL5 resulted in T cell deficiency but the expansion of myeloid progenitors (74).

Among CXC-chemokines, CXCL1 and CXCL2 are also known to suppress the *ex vivo* proliferation of myeloid progenitors by activation of CXCR2 (75, 76). The myelosuppressive activity of CXCR2 signaling was confirmed as mice lacking CXCR2 (*Cxcr2^−/−^*) have enhanced expansion of myeloid progenitors in BM and neutrophilia in peripheral blood (76–78). Although *Cxcr2^−/−^* stem/progenitor cells showed initial expansion *in vivo*, these cells had exhausted self-renewal capacity in a serial transplantation assay (78). However, *Cxcr2^−/−^* mice displayed comparable numbers and cytokine responsiveness of myeloid progenitors in BM, but abnormal retention of mature neutrophils (79). In another elegant study, Mei, J. et al. (80) showed that *Cxcr2^−/−^* or *Cxcl5^−/−^* mice had IL-17A/G-CSF and commensal bacteria dependent-neutrophil hyperplasia in BM and mild neutrophilia in peripheral blood during steady state. Using chimeric mice, the authors delineate that the loss of CXCR2 in the hematopoietic compartment contributed to enhanced granulopoiesis while enterocytes and BM resident cells supplied CXCL5 under basal conditions (80). Recently, we examined the role of CXC chemokine in context of emergency granulopoiesis. Contrastingly, we showed that mice deficient in CXCL1 (*Cxcl1^-/-^*) displayed reduced blood neutrophilia and recruitment to lungs and a corresponding defect in generation of neutrophils in BM (81). Using multi-parametric flow cytometry, *Cxcl1^-/-^* mice showed reduced numbers of hematopoietic stem cells and granulocyte-monocyte progenitors, which can be corrected by exogenous administration of other *CXCR2* ligands such as CXCL2, CXCL5 (81).

## Interleukins

IL-6 is the most studied interleukin in the context of hematopoiesis. Although mice deficient in IL-6 (*Il-6^-/-^*) have no apparent deficit in neutrophil number during the basal state (82), they displayed reduced neutrophilia and neutrophil response during *Listeria* and *Candida* infections (83, 84). Since enhanced granulopoiesis in *Gcsf^-/-^ and Gmcsf^-/-^* mice were associated with elevated IL-6 levels (51), the usage of mice with triple deficiency of IL-6, G-CSF, GM-CSF implicated IL-6 as a rescue cytokine that can take over granulopoiesis in the absence of classical granulopoietic cytokines, G-CSF and GM-CSF (85). Since these triple knock-out mice died shortly after birth, an *in vitro* granulopoiesis using embryonic fibroblasts revealed an additive effect for IL-6 (85). The neutrophil promoting activity of medium conditioned by *Gcsf^-/-^/Gmcsf^-/-^/Il-6^-/-^* fibroblasts was reduced by 40% compared to *Gcsf^-/-^/Gmcsf^-/-^* fibroblasts (85). Zhao et al. (29) and Schurch et al. (86) demonstrated that HSPCs and MSC produced IL-6 in response to inflammatory signals that stimulate HSC mobilization and myeloid differentiation in neutropenia or to promote clearance of the infection. More recently, it has been shown that TLR4 signaling-derived IL-6 promotes emergency granulopoiesis by phosphorylating C/EBP-α/β (87).

The role of IL-3 in emergency granulopoiesis is discrepant (88, 89). Innate response activator (IRA) B cells are the main producers of IL-3 during polymicrobial sepsis, and it is essential for emergency hematopoiesis, but dampens host survival benefit (89). In contrast, the specific depletion of IRA B cells in sepsis was detrimental as it leads to loss of GM-CSF signaling, increase in bacterial burden, and elicits cytokine storm (90). Unlike IL-6 and IL-3, the involvement of the IL-1 family and their receptor IL-1R in hematopoiesis have not been explored extensively until recently. Mice deficient in IL-1R (*Il-1r^-/-^*) have reduced proliferation of HSCs, MPPs, and GMP during alum-induced inflammatory granulopoiesis (91). Indeed, Pietras et al. (92) demonstrated that acute exposure of IL-1 enhanced the HSC proliferation and differentiation along the myeloid lineages through activation of transcription factor PU.1, but its chronic exposure impaired HSC functions and self-renewal capacity. IL-27, one of the IL-6/IL-12 family cytokines, has been recently shown to specifically promote the expansion and differentiation of LSK cells, particularly MPPs, into myeloid lineages in synergy with stem cell factor (93). Similarly, IL-27 transgenic mice displayed enhanced myelopoiesis and impaired development of B cell lineages (94). These results suggest that IL-27 is one of the limited cytokines that play a role in HSC regulation.

## Interferons

Type I IFNs (IFN-α/β) and Type II IFNs (IFN-γ) are pro-inflammatory cytokines that regulate early hematopoiesis. Early reports using a model of virus-induced transient pancytopenia revealed that mice lacking α/β (*IFN-α/βR^-/-^*) or γ (*IFN-γR^-/-^*) receptors have a different degree of BM cellularity (95). Indeed, during virus-induced pancytopenia, BM cells (specifically pluripotent and committed progenitors) were substantially lost in WT and *IFN-γR^-/-^*, but not in *IFN-α/βR^-/-^* mice (95). Later, two elegant reports confirmed that IFN-α stimulates the exit and proliferation of HSCs in two different models (96, 97). Essers et al. (96) reported that the *in vivo* treatment of IFN-α enhanced the HSC proliferation in mice and HSCs deficient in IFN-α/βR were unresponsive to the treatment. Similarly, Sato et al. (97) discovered mice deficient in interferon response factor 2 (*Irf2^-/^* ^-^), a transcriptional suppressor of IFN-α signaling, have an enhanced proportion of proliferating HSCs and *Irf2^-/^* ^-^ HSCs have accelerated cell division and failed to generate hematopoietic cells. Unlike Type I IFNs, the role of IFN-γ appears to be controversial in HSC proliferation. IFN-γ is sufficient to induce HSCs proliferation *in vivo* and HSCs from *Ifnγr^-/-^* mice have a reduced rate of proliferation (94). In stark contrast, IFN-γ has been shown to impair HSC proliferation and restoration during viral infection through inducing SOCS1 expression, thereby suppressing STAT5 phosphorylation (98). Moreover, the anti-proliferative activity of IFN-γ was shown to be dependent on integrin β3 signaling as it promotes serine phosphorylation of STAT1 in HSCs (99). Additionally, de Bruin et al. demonstrated that IFN-γ determined the fate of GMPs to monocytes over neutrophils during viral infection as IFN-γ enhanced the expression of PU.1 and IRF8 but dampened STAT3 phosphorylation (100).

## Reactive oxygen species

ROS content of HSCs has been known to regulate their self-renewal, migration, and development (101–105). While ROS-low-HSCs are long-term repopulating and low cell cycling, ROS-high-HSCs are short-term repopulating, cell cycling, and differentiating (102, 103). Recently, the cell extrinsic effect of ROS on hematopoiesis has been explored in thioglycolate and infection-elicited granulopoiesis (106, 107). BM myeloid (GR-1^+^) cells produced NADPH oxidase-dependent ROS, which regulates the expansion of GMPs *via* a PTEN oxidation-dependent manner during *E. coli*-induced emergency granulopoiesis (107). Later, another study by the same group demonstrated that ROS-producing BM GR-1^+^ cells are also critical for neutrophil homeostasis in sterile inflammation-induced granulopoiesis (106). However, the contribution of pathogen sensing into ROS production by the BM GR-1^+^ niche and its cell specific proliferation of GMPs, but not MEP and CEP as shown by these studies needs further investigation.

## Regulation of emergency granulopoiesis

At the molecular level, several transcription factors are involved in cell fate decision, differentiation, and proliferation.

## CCAAT/Enhancer-binding proteins (C/EBPs)

Several studies have shown that transcription factors CCAAT/enhancer-binding proteins (C/EBPs) -α, -β, and -ϵ have major regulatory roles in neutrophil granulopoiesis (8, 108, 109). C/EBP-α, the prototypical basic-region leucine zipper (bZIP) transcription factor, is mostly expressed in myeloid cells within the hematopoietic system and known to regulate steady state granulopoiesis (17, 18). Mice deficient in C/EBP-α (*C/EBP-α^-/-^*) displayed impaired steady-state granulopoiesis, as they selectively lacked mature granulocytes but retain monocytes (18). Furthermore, these mice were unresponsive to exogenous IL-6 and G-CSF since expression of IL-6 and G-CSF receptors were compromised with loss of C/EBP-α (18, 110). *C/EBP-α^-/-^* fetal liver progenitors displayed high plating capacity for HSPCs but a striking block in differentiation of MPP into bipotent GMPs both *in vivo* and *in vitro* (111). Mechanistically, C/EBP-α negatively regulates the expression of c-Myc, a cell proliferative factor, through an E2F binding site, thereby altering myeloblasts differentiation to CMPs (112). Moreover, C/EBP-α directly causes proliferation arrest by inhibiting cyclin-dependent kinase 2 (*Cdk2*) and *Cdk4* (113). C/EBP-α also decides cell fate as it directs a myeloid gene expression program through interacting factors involved in the expression of *Il-6r*, *Csf3r*, *Gfi-1*, *Irf-8*, and *Klf5* (110, 114–116). For instance, the expression of C/EBPα in HSCs or *in vivo* activation of C/EBPα in mice resulted in increases in myeloid progenitors and granulocytes but concomitant decreases in erythroid and/or non-myeloid progenitors (117, 118). Further, the conditional silencing of C/EBPα in HSPCs led to the loss of expression of myeloid genes but aberrant expression of T cell-related genes such as *Cd7* and *Lck* (119). C/EBP-ϵ regulates terminal differentiation of neutrophil development (120). Although *C/EBP-ϵ^-/-^* mice have no defect in generation of all blood cell lineages, they failed to develop functional neutrophils (121).

Conversely, C/EBP-β is upregulated in hematopoietic sub-populations during infectious or inflammatory states and orchestrates emergency granulopoiesis (9, 33, 122, 123). C/EBP-β can be induced in *C/EBP-α^-/-^* cells *in vitro* with IL-3, GM-CSF stimulation, or transduction with G-CSF to generate neutrophils, suggesting the existence of C/EBP-α-independent pathways for granulocyte generation (110, 124). Hirai et al. (9) were first to show conclusively that a large number of mature granulocytes can be generated from *C/EBP-α^-/-^* progenitors following cytokine (G-CSF, GM-CSF, and IL-3) treatment. Following *C. albicans* or cytokine administration, the sustained upregulation of C/EBP-β, but not C/EBPs-α, -δ, and –ϵ, was observed in granulocyte progenitors, and *C/EBP-β^-/-^* progenitors exhibit impaired proliferation and differentiation during cytokine- or fungal–induced emergency granulopoiesis (9, 123). Unlike C/EBPα, C/EBP-β-mediated emergency granulopoiesis may be dependent on prolonged expression of c-Myc in progenitor cells (9). How pathogen sensing during systemic infection leads to switch from C/EBP-α to C/EBP-β to promote emergency granulopoiesis is unknown.

## STAT proteins

The exact mechanism of the switch from C/EBP-α-dependent steady state to C/EBP-β-mediated emergency granulopoiesis during the infectious state is poorly understood. However, the heightened level of granulocytic cytokines (G-CSF, GM-CSF, IL-6) and their interactions with receptors and cellular targets have revealed several other transcription factors that may act as a bridge between pathogen sensing and molecular translation during emergency granulopoiesis. For examples, the engagement of G-CSF signaling induces JAK-STAT pathways that mostly involve activation of STAT3 but also to a lesser extent STAT1 and STAT5. *Gcsfr* ^-/-^ mice displayed impaired activation of STAT3 and STAT5 and a defect in G-CSF-induced granulocytic differentiation *in vivo* and *in vitro* (125). Furthermore, STAT3 directly regulates expression of C/EBP-β in Gr-1+ granulocytes in *L. monocytogenes*-induced granulopoiesis, and STAT3 favors increase in occupancy of C/EBP-β over C/EBP-α to the c-Myc promoter during G-CSF-induced granulopoiesis (126). However, mice harboring STAT3 deletion in hematopoietic progenitors exhibited enhanced output of granulocyte precursors and functional neutrophils in G-CSF-induced granulopoiesis, providing direct evidence of a STAT3-dependent negative feedback loop (127). Mechanistically, deficiency of STAT3 resulted in impaired induction of suppressor of cytokine signaling (SOCS3) which otherwise would act as negative regulator of JAK catalytic activity during G-CSF-induced granulopoiesis (127). Similarly, *Socs3^-/-^* neutrophilic granulocytes displayed enhanced STAT3 activity *in vitro* (128) and *Socs3^-/-^* mice develop neutrophilia when injected with G-CSF (128, 129). The GM-CSF receptor pathways also participate in emergency granulopoiesis, and it is mediated through STAT5A and STAT5B (50, 130). *Stat5a/b^-/-^* mice exhibit defects in GMP generation and neutrophil survival during GM-CSF-induced granulopoiesis or 5FU-mediated myelosuppression (130).

## Other myeloid transcription factors

Besides C/EBPs and STATs, *Ets* transcription factors PU.1, Runt-related transcription factor 1 (Runx1), zinc finger transcription factor (Gfi-1), lymphoid enhancer-binding factor 1 (LEF1), Interferon regulatory factor 8 (IRF8), and homeobox gene-A10 (HOXA10) are other important transcription factors regulating granulopoiesis. PU.1 is critical for development of all hematopoietic lineages (131) and the expression level of PU.1 determines lineage choice as low concentration of PU.1 allows C/EBP-α to favor granulocytes generation over monocytes from myeloblasts during steady-state granulopoiesis (132). One study reported that *Runx1^-/-^* mice exhibit a mild myeloproliferative phenotype with enhanced neutrophilia and expanded myeloid progenitors (133), while another study demonstrated that deletion or dominant inhibition of Runx1 reduces C/EBP-α and favors monopoiesis over granulopoiesis (134). Gfi1 antagonizes PU.1 (135) and is essential for steady-state and emergency granulopoiesis, and consequently *Gfi1^-/-^* mice are highly susceptible to bacterial infection (136). LEF1 directly controls C/EBP-α expression and mediates generation and differentiation of neutrophils (137). IRF8 influences the expression of Fanconi DNA repair proteins (i.e., FNCC and FNCF) which are critical for genomic stability during S phase (138, 139). *Irf8^-/-^* mice displayed decreased FAS sensitivity in myeloid progenitors and sustained granulocyte generation during alum-induced emergency granulopoiesis (140). Mice deficient in Fanconi-C were neutropenic and exhibited impaired HSC survival and function in alum-induced granulopoiesis (138). Like SOCS3 and IRF8, HOXA10 is a negative regulator of emergency granulopoiesis as *Hoxa10^-/-^* mice develop uncontrolled neutrophilia associated with heightened activity of Triad1, an anti-proliferative E3 ubiquitin ligase (141, 142). More recently, secretory leukocyte protease inhibitor (SLPI), a natural repressor of neutrophil elastase, has been shown to regulate the expression of several transcription factors in G-CSF-triggered emergency granulopoiesis and it is severely reduced in congenital neutropenic patients (143). Transducing SLPI-shRNA into human BM CD34+ progenitors and leukemic NB4 cells, Klimenkova et al. demonstrated that SLPI regulates G-CSF-induced granulopoiesis by regulating NFκB levels, ERK1/2 phosphorylation and activation of LEF-1, and c-Myc-dependent signaling pathways (143). Recently, Danek et al. demonstrate the β-Catenin-TCF/LEF directly regulates G-CSF receptor, thereby consequently controlling steady and emergency granulopoiesis (144).

## Microbiota

As crosstalk between host microbiome and immunity is well recognized, a large body of evidence suggests that gut microbiota can directly regulate neutrophil homeostasis and contribute to priming host immune responses. Antibiotic-treated and germ-free mice exhibit reduced numbers of granulocyte progenitors in BM and spleen, and a decline in blood neutrophils (80, 145–149). Mice replenished and enriched with gut microbiota exhibit restored granulopoiesis (145, 150, 151). Microbiota-derived products such as peptidoglycan or lipoprotein can make their way into the BM niche, thereby allowing HSPCs and myeloid precursors to detect them and initiate hematopoiesis directly (148, 151–153). Another possibility is that HSPCs can exit the BM, patrol peripheral organs or even reside there for few days, and encounter gut microbiota-derived pathogens/their products before re-entering the BM (14, 15). On other hand, gut microbial products such as LPS can activate TLR4/MyD88 pathways to induce IL-17A and G-CSF, thereby indirectly activating granulopoiesis (145). Granulopoiesis in germ-free (GF) mice is also dependent on TLR signaling; as *Myd88^-/-^/Ticam* ^-/-^ GF mice failed to restore microbiota-driven granulopoiesis when transferred with microbiota or serum from colonized mice (151). The malnourished host has impaired hematopoiesis, and supplementation of immunobiotics such as *Lactobacillus rhamnosus* CRL1505 in the host restored the granulopoiesis and improved immune responses (154). Thus, it is tempting to speculate that microbiota may have educated the hematopoietic system to respond to peripheral demands along a continuum, and manipulation of microbiota or immunobiotics could improve host immunity in neutropenic patients.

## Macrophages

The macrophage’s inability to clear apoptotic neutrophils regulates steady-state granulopoiesis (155–157). After phagocytosis of aged neutrophils, macrophages secrete less IL-23 thereby curbing down IL-17/G-CSF axis and steady-state granulopoiesis (155). Mice harboring a conditional deletion of the anti-apoptotic gene cellular FLICE-like inhibitory protein (c-FLIP) in myeloid cells (*c-FLIP^f/f^LysM-Cre*) lacks macrophages specifically the in marginal zone and BM stroma (156). *c-FLIP^f/f^LysM-Cre* mice exhibited delayed clearance of circulating neutrophils and developed severe neutrophilia in a G-CSF-dependent but not IL-17-dependent manner (156). Mechanistically, the engulfment of senescent neutrophils activates liver x receptors (LXR) in macrophages which directly suppress the IL-23/IL-17/G-CSF granulopoietic cascade (157). Recently, mast cells have been shown to enhance macrophage phagocytosis, and mice lacking mast cells (Kit*^W‐sh^*) exhibited elevated IL-17/G-SCF activity, aberrant intramedullary myelopoiesis, and peripheral blood neutrophilia (158).

## Non-hematopoietic BM stromal cells

With recent advancements in reporter mouse models, BM chimeras, imaging, and cell sorting techniques, the presence of the non-hematopoietic cellular compartment has been mapped out and appreciated as a critical regulator of granulopoiesis and immunity (159). BM endothelial cells express *TLR4* and *MyD88* at the basal state and respond with heightened G-CSF expression upon LPS stimulation (36). TLR4 signaling confined to the non-hematopoietic compartment only is sufficient to promote LPS-induced granulopoiesis (35). In particular, VCAM^+^CD146^low^ BM stromal fibroblasts produce IL-6 which contributes to expansion of GMPs at the expense of erythropoiesis during *Toxoplasma gondii* infection (160). Similarly, BM CXCL12-abundant reticular (CAR) cells secrete IL-6 in response to IFN-γ by CD8 T cells during viral infection, and IL-6 in turn favors HSPCs differentiation toward myeloid lineages (86).

## Special notes on neutrophil circadian rhythms

**Figure 4:**
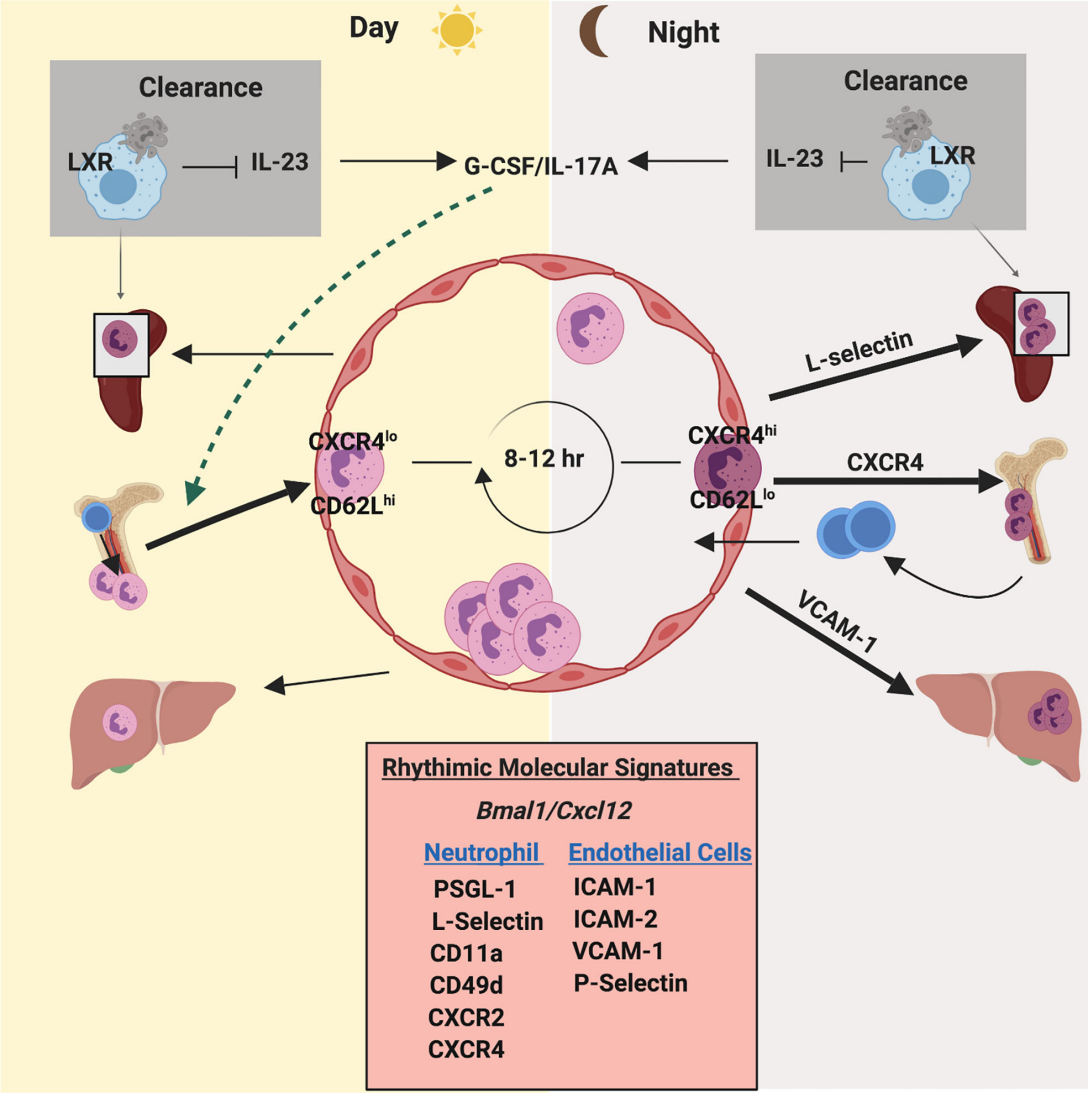
Circadian rhythm regulates neutrophil homeostasis and function. Neutrophils exhibit an oscillation in numbers in blood and tissues, which coincides with daily circadian rhythms. Neutrophil numbers in blood peak after few hours of initiation of light and then drop during the light phase. Under the influence of clock genes (such as Bmal1) and granulopoietic cytokines (mainly G-CSF), CXCL12/CXCR4 regulates daily release and retention of neutrophils. Furthermore, a circadian fluctuation of the expression of CD62L and CXCR4 on the surface of neutrophils dictates their counts in blood during steady state. CD62L^high^CXCR4^low^ neutrophils enter circulation during the light phase. After 8-12 hours, aged neutrophils (CD62L^low^CXCR4^high^) enter the bone marrow for clearance coinciding with daily circadian rhythms. Additionally, oscillatory expression of pro-inflammatory and adhesive molecules on the endothelium and on neutrophils exhibit a robust circadian rhythmicity during neutrophil release and egress.

Circadian control of the immune system is an emerging concept of the regulation of immune homeostasis (161) (**Figure 4**). A recent study has shown that, like HSCs (162) and monocytes (163), circulating neutrophils also follow daily circadian fluctuations, as they age in peripheral blood and re-enter the BM for clearance (164). Mechanistically, daily aged circulating neutrophils are characterized by expression of low levels of L-selectin (CD62L) but high levels of CXCR4, guiding their way back to the BM within 12 hours (164). Using an organism-wide circadian screening approach, an elegant study has identified circadian expression of trafficking molecules (ICAM-1, VCAM-1, CD11a, CD49d, CXCR4) for neutrophils and other leukocytes, regulating the rhythmic emigration of leukocytes from blood (165). More specifically, chronopharmacological targeting of these migratory molecules has revealed that CXCR4, L-selectin, and ICAM-1 govern rhythmic neutrophil migration to the bone marrow, spleen, and liver respectively (165). Following an acute LPS exposure, the blood counts of neutrophils and other leukocytes exhibited a dramatic drop but their time-of-day differences between day and night were preserved, extending the notion of neutrophil rhythmic oscillation to the inflammatory state (165). Although circadian control of neutrophil oscillation is still in its infancy, we can speculate that it is a critical mechanism of immune sentinels to maintain neutrophil homeostasis and determine the strength of the immune response during infection and inflammation.

## Trained immunity

Recently, several epidemiological studies have shown that vaccination or prior exposure to a disease provide non-specific heterologous protection to non-related diseases through epigenetic imprinting of immunological memory of innate cells, a phenomenon termed as “Trained immunity’ or “Innate immune memory” (166). Trained immunity is orchestrated by epigenetic imprinting, which involves histone modifications, as well as, transcriptional and functional reprogramming of innate and non-immune cells following vaccination and infection (167). Kalafati et al. have shown that beta-glucan-induced trained immunity is associated with transcriptomic and epigenetic imprinting of granulopoiesis, which has an anti-tumor effect (168).

## Human vs mouse granulopoiesis

Our current knowledge of granulopoiesis largely depends on data from murine models. However, we recognize there are significant differences in the maturation, migration, heterogeneity, and function of human and mouse neutrophils. Neutrophils accounts for up to ∼70% of all peripheral leukocytes in humans and ∼10–20% in mice (169). Furthermore, human and mouse neutrophils differ in neutrophil molecules (selectins, FcαRI, serine proteases) (170–172), expression of cytokines (IL-10, IL-17) (173), activation pathways of ROS (174), and cytotoxic granular content (defensins, bactericidal enzymes) (175) that can collectively alter the overall biology and immunology of these cells between species. On the other hand, a recent study utilizing single-cell transcriptomics to investigate human and mouse lung cancers revealed conservation of neutrophil subsets between species (176, 177).

## Concluding remarks and future directions

In summary, it is very clear now that neutrophil homeostasis during the infectious state is a complex and tightly regulated process. Neutrophil homeostasis involves the sensing of pathogens or other stimuli by hematopoietic and non-hematopoietic cells (TLR/MyD88 pathways), the release of growth factors (G-CSFs, IL-6, CXCs) to activate several transcription machineries, and the ultimate instruction to activate dormant HSC and generate neutrophils in the BM (C/EBP-β, STATs), all gearing to mount a protective hematopoietic immune response. C/EBP-β mediates emergency granulopoiesis and C/EBP-α regulates steady-state granulopoiesis.

Accumulating data from several preclinical models have deepened our knowledge regarding mechanisms of neutrophil homeostasis. In a few instances, however, they have generated conflicting data and authors have sometimes postulated different and contradictory mechanisms of neutrophil homeostasis. These discrepancies can be explained by differences in experimental model (infection or cytokine-induced), types and inoculum of pathogens (bacterial vs. fungal), route of infections, timing of analysis, endpoints to measure granulopoiesis (BM vs. peripheral blood output, host survival vs. recruitment), and even composition of microbiota. We suggest that these discrepancies can be resolved by the development of appropriate experimental models to test clearly defined endpoints to study emergency granulopoiesis.

It is now obvious that the hematopoietic system has emerged to detect invading pathogens in a very fast and versatile way. A diverse spectrum of microbial messaging to the bone marrow is in place. For instance, direct pathogen sensing by HSC and myeloid progenitors allows the hematopoietic system to rapidly respond with early granulopoiesis to control imminent infection. How this pathway of direct activation differs from indirect activation of HSPCs in terms of conversion of pathogen signal to molecular cues during granulopoiesis is largely unknown. Does waking up HSC with TLR agonists or PAMPs have clinical use? Furthermore, future studies should be directed to investigate the relative contributions and differences of direct versus indirect HSPC activation. With the discovery of ECs regulating the emergency granulopoietic response, it is possible that vasculature linings ECs are a critical layer of the hematopoietic system that convert systemic spreading pathogen signals to molecular cues. Additionally, various bone marrow stromal cells, macrophages, and aged neutrophils appear to control granulopoiesis. Is there any crosstalk between hematopoietic and resident cells during granulopoiesis? What will be their contributions? Do they initiate distinct signaling mechanisms? An emerging challenge to hematologists and immunologists would be to solve how these diverse pathogen signals, picked up by different cells, are converted by HSC to launch a mechanism of emergency granulopoiesis.

Growth factors, cytokines, and chemokines activate HSCs, instruct lineage choice, and regulate the generation of neutrophils, thereby making them excellent molecular targets for therapeutics. G-CSF and GM-CSF are already in use for clinical applications to regenerate myeloid cells. However, decades of preclinical studies have demonstrated that these signaling pathways are extremely redundant. For instance, IL-6 can surrogate the function of all three colony-stimulating factors during emergency granulopoiesis. Experimental models with gain and loss of function of these specific pathways are warranted to understand their specific role. Thus, understanding the common cellular targets of these molecular messengers would be of great interest for therapeutic efficiency.

In addition, we feel that there is limited knowledge on key cellular, molecular, and environmental players that regulate emergency granulopoiesis. Several outstanding questions remain unanswered. What are the mechanisms in place to prevent an uncontrolled granulopoietic response? Could dysregulation of the granulopoietic response or direct HSC activation contribute to BM failure and development of hematologic diseases? How do we translate our findings from preclinical studies into human infectious and hematologic diseases? A systems biology and single-cell approach may be needed to understand how hematopoietic systems integrate molecular signals into a single and unique granulopoietic response. The evolving role of the host microbiome in shaping and priming the hematopoietic response is another promising area to explore.

A better understanding of sensors, mediators, and regulators of emergency granulopoiesis may inspire novel therapeutic approaches for neonatal, pediatric, and adult infectious diseases. Signaling pathways used during emergency granulopoiesis could be examined and exploited for development of therapeutic targets for hematologic as well as malignant diseases.

## Author contributions

SP wrote the draft along with LG and LJ. DJ and SJ edited the draft. All authors contributed to the article and approved the submitted version.

## Acknowledgments

We would like to acknowledge members of the Center for Lung Biology and Diseases, including Tirumalai Rangasamy, Joseph DeCorte, John T. Le and Dinesh Bhattarai, for helpful discussions. This work is supported by R01AI157353 (SJ), R01AI140500 (SJ) and P20GM130555 (SJ) from the National Institute of Health.

## Conflict of interest

The authors declare that the research was conducted in the absence of any commercial or financial relationships that could be construed as a potential conflict of interest.

## Publisher’s note

All claims expressed in this article are solely those of the authors and do not necessarily represent those of their affiliated organizations, or those of the publisher, the editors and the reviewers. Any product that may be evaluated in this article, or claim that may be made by its manufacturer, is not guaranteed or endorsed by the publisher.
